# 4-Hy­droxy-2-methyl-3,4-diphenyl­cyclo­pent-2-en-1-one

**DOI:** 10.1107/S1600536810019112

**Published:** 2010-05-26

**Authors:** Abdul Rauf Raza, Aeysha Sultan, M. Nawaz Tahir

**Affiliations:** a Department of Chemistry, University of Sargodha, Sargodha, Pakistan; b Department of Physics, University of Sargodha, Sargodha, Pakistan

## Abstract

The asymmetric unit of title compound, C_18_H_16_O_2_, contains two mol­ecules with slightly different conformations. In the first mol­ecule, the two phenyl rings make dihedral angles of 84.98 (11)° and the five-membered ring makes dihedral angles of 84.80 (12) and 73.00 (12)° with the phenyl rings; the corresponding angles for the second mol­ecule are 86.74 (11), 81.20 (13) and 71.36 (12)°. O—H⋯O hydrogen bonds between the hy­droxy and carbonyl groups are a feature of the crystal packing, which results in chains extending parallel to [100]. Weak C—H⋯O and C—H⋯π inter­actions are also observed.

## Related literature

For background to the biological applications of steroids, see: Berger *et al.* (1996 ▶); Yamada (2002 ▶). For related structures, see: Sher *et al.* (2007 ▶); Katritzky *et al.* (1999 ▶).
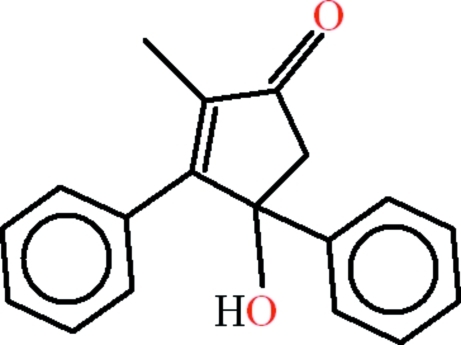


## Experimental

### 

#### Crystal data

C_18_H_16_O_2_
*M* *_r_* = 264.31
Orthorhombic, 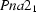

*a* = 12.5098 (4) Å
*b* = 13.1453 (5) Å
*c* = 17.5292 (7) Å
*V* = 2882.59 (18) Å^3^
*Z* = 8
Mo *K*α radiation
μ = 0.08 mm^−1^
*T* = 296 K
0.34 × 0.24 × 0.20 mm

#### Data collection

Bruker Kappa APEXII CCD diffractometer
Absorption correction: multi-scan (*SADABS*; Bruker, 2005 ▶) *T*_min_ = 0.979, *T*_max_ = 0.988
14465 measured reflections
3529 independent reflections
2258 reflections with *I* > 2σ(*I*)
*R*_int_ = 0.044

#### Refinement

*R*[*F* ^2^ > 2σ(*F* ^2^)] = 0.048
*wR*(*F* ^2^) = 0.115
*S* = 0.99
3529 reflections
365 parameters
1 restraint
H-atom parameters constrained
Δρ_max_ = 0.18 e Å^−3^
Δρ_min_ = −0.21 e Å^−3^

### 

Data collection: *APEX2* (Bruker, 2007 ▶); cell refinement: *SAINT* (Bruker, 2007 ▶); data reduction: *SAINT*; program(s) used to solve structure: *SHELXS97* (Sheldrick, 2008 ▶); program(s) used to refine structure: *SHELXL97* (Sheldrick, 2008 ▶); molecular graphics: *ORTEP-3 for Windows* (Farrugia, 1997 ▶) and *PLATON* (Spek, 2009 ▶); software used to prepare material for publication: *WinGX* (Farrugia, 1999 ▶) and *PLATON*.

## Supplementary Material

Crystal structure: contains datablocks global, I. DOI: 10.1107/S1600536810019112/wm2348sup1.cif

Structure factors: contains datablocks I. DOI: 10.1107/S1600536810019112/wm2348Isup2.hkl

Additional supplementary materials:  crystallographic information; 3D view; checkCIF report

## Figures and Tables

**Table 1:** Hydrogen-bond geometry (Å, °)

*D*—H⋯*A*	*D*—H	H⋯*A*	*D*⋯*A*	*D*—H⋯*A*
O1—H1⋯O2^i^	0.82	2.03	2.823 (3)	164
O3—H3*A*⋯O4^ii^	0.82	2.06	2.861 (3)	164
C2—H2⋯O4^ii^	0.93	2.54	3.430 (5)	162
C13—H13⋯O3	0.93	2.58	3.453 (4)	157
C20—H20⋯O2^iii^	0.93	2.60	3.478 (5)	158
C31—H31⋯O1^iv^	0.93	2.59	3.478 (5)	161

Symmetry codes: (i) 
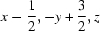
; (ii) 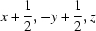; (iii) 
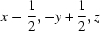
; (iv) 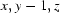; (v) 
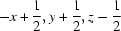
.

**Fig. 1.**
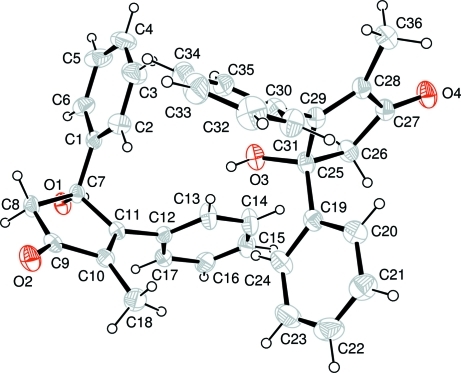
View of the title compound with the atom numbering scheme. Displacement ellipsoids are drawn at the 50% probability level. H-atoms are shown by small circles of arbitrary radius.

**Fig. 2.**
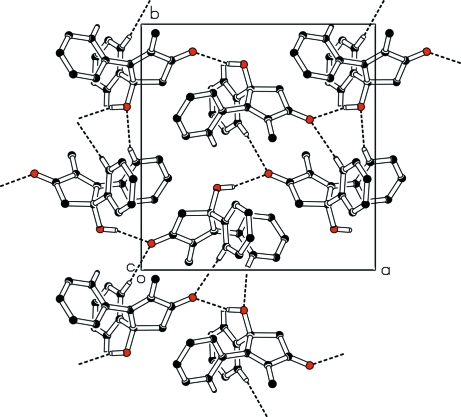
The partial packing (PLATON; Spek, 2009) which shows that molecules form polymeric chains extending along the b-axis.

## supplementary crystallographic information

### Comment

Steroids belong to a rich class of secondary metabolites with enormous biological applications, e.g. in the treatment of brain tumors (Yamada, 2002). They are effective against cancer (especially of breast, uterus and prostrate) and also in therapeutic treatment of AIDS symptoms (Berger *et al.*, 1996). The title compound (I, Fig. 1) has been synthesized as a D-ring precursor for the steroidal nucleus. The presence of a chiral center and the two phenyl rings makes it a novel substrate to synthesize a variety of steroids with different substituents on the D-ring.

Related crystal structures of 4-hydroxy-3,4-diphenyl-2-cyclopenten-1-one, (II), (Katritzky *et al.*, 1999) and ethyl 4-hydroxy-3,4-diphenyl-cyclo-2-penten-1-one-2-carboxylate, (III), (Sher *et al.*, 2007) have been published. The title compound differs from (II) and (III) due to substitution at the five membered ring.

The asymmetric unit of title compound consist of two molecules which differ slightly from each other. The phenyl rings A (C1—C6) and B (C12—C17) are attached to the five-membered ring C (C7—C11) which is nearly planar with a r. m. s. deviation of 0.0505 Å from the least-squares plane. The values of the dihedral angles between A/B, A/C and B/C are 84.98 (11)°, 84.80 (12)° and 73.00 (12)°, respectively. The other molecule consists of phenyl rings D (C19—C24), E (C30—C35) and the five-membered ring F (C25—C29). Here the five membered ring deviates by 0.0491 Å from the least-squares plane. The dihedral angles between D/E, D/F and E/F are 86.74 (11)°, 81.20 (13)° and 71.36 (12)°, respectively.

The packing of the molecules is characterised by intermolecular O—H···O hydrogen bonding between the hydroxy donor groups and the C═O acceptor groups, leading to the formation of chains extending parallel to [100] (Table 1, Fig. 2). Weak C—H···π and intramolecular π···π interactions are also observed. The five-membered and phenyl rings attached to the hydroxy containing C-atom show π···π interactions with separation in the range 3.767 (3)-3.779 (3) Å, whereas the five membered and other phenyl ring show a separation in the range 4.069 (3)-4.080 (3) Å.

### Experimental

Alcoholic KOH (0.6 g, 11 mmol) was added drop-wise to a well stirred hot solution of butanone (3.4 g, 0.06 mol) and benzil (2.1 g, 10 mmol) in EtOH (25 mL) and refluxed for 30 minutes. The resulting reaction mixture was cooled to 278 K in an ice bath and extracted with n-hexane (3 × 25 mL). The extract was dried over anhydrous Na_2_SO_4_ and concentrated under reduced pressure. The resulting solution was kept at room temperature for 48 h to afford colorless prisms.

### Refinement

All Friedel pairs were merged. Although H atoms were discernible in difference Fourier maps, they were positioned geometrically with (C-H = 0.93–0.97 and O-H = 0.82 Å) and refined as riding with *U*_iso_(H) = x*U*_eq_(C), where x = 1.5 for methyl and hydroxy H-atoms and x = 1.2 for all other H atoms.

### Crystal data

**Table:**

C_18_H_16_O_2_	*F*(000) = 1120
*M**_r_* = 264.31	*D*_x_ = 1.218 Mg m^−^^3^
Orthorhombic, *P**n**a*2_1_	Mo *K*α radiation, λ = 0.71073 Å
Hall symbol: P 2c -2n	Cell parameters from 1864 reflections
*a* = 12.5098 (4) Å	θ = 2.3–28.0°
*b* = 13.1453 (5) Å	µ = 0.08 mm^−^^1^
*c* = 17.5292 (7) Å	*T* = 296 K
*V* = 2882.59 (18) Å^3^	Prism, colorless
*Z* = 8	0.34 × 0.24 × 0.20 mm

### Data collection

**Table:**

Bruker Kappa APEXII CCD diffractometer	3529 independent reflections
Radiation source: fine-focus sealed tube	2258 reflections with *I* > 2σ(*I*)
graphite	*R*_int_ = 0.044
Detector resolution: 7.50 pixels mm^-1^	θ_max_ = 27.9°, θ_min_ = 2.3°
ω–scans	*h* = −16→15
Absorption correction: multi-scan (*SADABS*; Bruker, 2005)	*k* = −14→17
*T*_min_ = 0.979, *T*_max_ = 0.988	*l* = −21→23
14465 measured reflections	

### Refinement

**Table:**

Refinement on *F*^2^	Primary atom site location: structure-invariant direct methods
Least-squares matrix: full	Secondary atom site location: difference Fourier map
*R*[*F*^2^ > 2σ(*F*^2^)] = 0.048	Hydrogen site location: inferred from neighbouring sites
*wR*(*F*^2^) = 0.115	H-atom parameters constrained
*S* = 0.99	*w* = 1/[σ^2^(*F*_o_^2^) + (0.0593*P*)^2^] where *P* = (*F*_o_^2^ + 2*F*_c_^2^)/3
3529 reflections	(Δ/σ)_max_ < 0.001
365 parameters	Δρ_max_ = 0.18 e Å^−^^3^
1 restraint	Δρ_min_ = −0.21 e Å^−^^3^

### Special details

**Table:**

Geometry. Bond distances, angles etc. have been calculated using the rounded fractional coordinates. All su's are estimated from the variances of the (full) variance-covariance matrix. The cell esds are taken into account in the estimation of distances, angles and torsion angles
Refinement. Refinement of *F*^2^ against ALL reflections. The weighted *R*-factor *wR* and goodness of fit *S* are based on *F*^2^, conventional *R*-factors *R* are based on *F*, with *F* set to zero for negative *F*^2^. The threshold expression of *F*^2^ > σ(*F*^2^) is used only for calculating *R*-factors(gt) *etc*. and is not relevant to the choice of reflections for refinement. *R*-factors based on *F*^2^ are statistically about twice as large as those based on *F*, and *R*- factors based on ALL data will be even larger.

### Fractional atomic coordinates and isotropic or equivalent isotropic displacement parameters (Å^2^)

**Table:**

	*x*	*y*	*z*	*U*_iso_*/*U*_eq_	
O1	0.43730 (14)	0.83610 (14)	0.23156 (15)	0.0463 (8)	
O2	0.71910 (14)	0.61202 (16)	0.24487 (17)	0.0565 (8)	
C1	0.40828 (19)	0.7000 (2)	0.13864 (19)	0.0390 (10)	
C2	0.4119 (3)	0.5988 (3)	0.1175 (2)	0.0577 (14)	
C3	0.3602 (3)	0.5670 (3)	0.0516 (3)	0.0757 (17)	
C4	0.3048 (3)	0.6343 (4)	0.0076 (3)	0.078 (2)	
C5	0.3014 (3)	0.7344 (4)	0.0279 (3)	0.0743 (19)	
C6	0.3533 (2)	0.7671 (3)	0.0928 (2)	0.0555 (14)	
C7	0.4671 (2)	0.7358 (2)	0.2095 (2)	0.0370 (10)	
C8	0.58990 (19)	0.7350 (2)	0.1973 (3)	0.0451 (13)	
C9	0.6291 (2)	0.6478 (2)	0.2462 (2)	0.0418 (10)	
C10	0.5418 (2)	0.6131 (2)	0.29581 (19)	0.0400 (10)	
C11	0.4522 (2)	0.6621 (2)	0.2763 (2)	0.0364 (10)	
C12	0.3469 (2)	0.6473 (2)	0.31338 (19)	0.0380 (10)	
C13	0.2870 (2)	0.5598 (2)	0.3005 (3)	0.0602 (14)	
C14	0.1906 (3)	0.5460 (3)	0.3375 (3)	0.0737 (16)	
C15	0.1529 (3)	0.6174 (3)	0.3867 (3)	0.0680 (16)	
C16	0.2094 (3)	0.7036 (3)	0.3992 (2)	0.0580 (14)	
C17	0.3062 (2)	0.7196 (3)	0.3627 (2)	0.0486 (11)	
C18	0.5604 (3)	0.5369 (3)	0.3565 (2)	0.0603 (14)	
O3	0.32467 (15)	0.33328 (14)	0.20448 (16)	0.0504 (9)	
O4	0.04405 (14)	0.10816 (16)	0.19042 (16)	0.0578 (9)	
C19	0.3572 (2)	0.1967 (2)	0.2955 (2)	0.0424 (11)	
C20	0.3643 (3)	0.0942 (3)	0.3135 (2)	0.0524 (14)	
C21	0.4182 (3)	0.0620 (3)	0.3771 (3)	0.0680 (16)	
C22	0.4655 (3)	0.1303 (4)	0.4252 (3)	0.0763 (18)	
C23	0.4582 (3)	0.2321 (4)	0.4091 (3)	0.077 (2)	
C24	0.4038 (3)	0.2651 (3)	0.3451 (3)	0.0597 (14)	
C25	0.2960 (2)	0.2323 (2)	0.2249 (2)	0.0407 (12)	
C26	0.1746 (2)	0.2301 (3)	0.2379 (3)	0.0473 (13)	
C27	0.1345 (2)	0.1439 (2)	0.1897 (2)	0.0423 (10)	
C28	0.2211 (2)	0.1101 (2)	0.1395 (2)	0.0407 (10)	
C29	0.3113 (2)	0.1595 (2)	0.1582 (2)	0.0383 (10)	
C30	0.4168 (2)	0.1482 (2)	0.1203 (2)	0.0406 (10)	
C31	0.4777 (3)	0.0610 (3)	0.1311 (2)	0.0570 (14)	
C32	0.5755 (3)	0.0508 (3)	0.0950 (3)	0.0737 (16)	
C33	0.6132 (3)	0.1264 (3)	0.0488 (3)	0.0720 (16)	
C34	0.5541 (3)	0.2128 (3)	0.0372 (2)	0.0657 (16)	
C35	0.4561 (3)	0.2232 (3)	0.0731 (2)	0.0495 (11)	
C36	0.2035 (2)	0.0339 (3)	0.0767 (2)	0.0549 (11)	
H1	0.37229	0.83885	0.23720	0.0694*	
H2	0.44895	0.55212	0.14727	0.0694*	
H3	0.36338	0.49889	0.03733	0.0908*	
H4	0.26950	0.61204	−0.03606	0.0940*	
H5	0.26397	0.78070	−0.00210	0.0893*	
H6	0.35112	0.83560	0.10579	0.0662*	
H8A	0.60733	0.72363	0.14405	0.0539*	
H8B	0.62148	0.79875	0.21358	0.0539*	
H13	0.31190	0.51047	0.26691	0.0725*	
H14	0.15113	0.48720	0.32866	0.0883*	
H15	0.08826	0.60711	0.41176	0.0817*	
H16	0.18311	0.75244	0.43268	0.0693*	
H17	0.34399	0.77943	0.37133	0.0582*	
H18A	0.50312	0.53975	0.39288	0.0903*	
H18B	0.62685	0.55134	0.38169	0.0903*	
H18C	0.56338	0.47018	0.33435	0.0903*	
H3A	0.38994	0.33785	0.20188	0.0757*	
H20	0.33191	0.04649	0.28184	0.0629*	
H21	0.42273	−0.00718	0.38764	0.0816*	
H22	0.50206	0.10802	0.46821	0.0916*	
H23	0.49005	0.27928	0.44142	0.0916*	
H24	0.39849	0.33448	0.33533	0.0717*	
H26A	0.15832	0.21841	0.29130	0.0566*	
H26B	0.14231	0.29382	0.22223	0.0566*	
H31	0.45260	0.00941	0.16270	0.0682*	
H32	0.61587	−0.00786	0.10233	0.0885*	
H33	0.67917	0.11914	0.02495	0.0865*	
H34	0.57969	0.26405	0.00554	0.0785*	
H35	0.41603	0.28192	0.06520	0.0591*	
H36A	0.27081	0.01691	0.05371	0.0824*	
H36B	0.15696	0.06259	0.03878	0.0824*	
H36C	0.17141	−0.02639	0.09744	0.0824*	

### Atomic displacement parameters (Å^2^)

**Table:**

	*U*^11^	*U*^22^	*U*^33^	*U*^12^	*U*^13^	*U*^23^
O1	0.0357 (10)	0.0371 (12)	0.0662 (18)	−0.0001 (8)	0.0019 (12)	0.0014 (12)
O2	0.0280 (11)	0.0599 (14)	0.0817 (18)	0.0063 (9)	−0.0022 (11)	0.0020 (14)
C1	0.0258 (14)	0.0482 (19)	0.043 (2)	−0.0009 (12)	0.0025 (14)	0.0055 (16)
C2	0.060 (2)	0.060 (2)	0.053 (3)	−0.0002 (16)	−0.0040 (18)	−0.004 (2)
C3	0.078 (3)	0.087 (3)	0.062 (3)	−0.014 (2)	0.001 (2)	−0.023 (3)
C4	0.049 (2)	0.136 (5)	0.050 (3)	−0.011 (2)	−0.006 (2)	−0.022 (3)
C5	0.058 (3)	0.117 (4)	0.048 (3)	0.024 (2)	−0.010 (2)	0.004 (3)
C6	0.0484 (19)	0.073 (2)	0.045 (3)	0.0121 (16)	0.0026 (18)	0.006 (2)
C7	0.0253 (13)	0.0386 (16)	0.047 (2)	0.0008 (11)	0.0029 (13)	0.0049 (16)
C8	0.0248 (14)	0.0534 (18)	0.057 (3)	−0.0038 (12)	0.0051 (15)	0.0062 (18)
C9	0.0273 (14)	0.0472 (17)	0.051 (2)	−0.0003 (12)	−0.0058 (14)	−0.0058 (16)
C10	0.0335 (15)	0.0425 (17)	0.044 (2)	0.0016 (12)	−0.0026 (14)	−0.0009 (16)
C11	0.0306 (15)	0.0367 (16)	0.042 (2)	−0.0015 (12)	−0.0013 (13)	−0.0026 (15)
C12	0.0329 (14)	0.0421 (16)	0.039 (2)	0.0000 (12)	0.0014 (13)	0.0024 (15)
C13	0.0487 (19)	0.047 (2)	0.085 (3)	−0.0106 (14)	0.018 (2)	−0.010 (2)
C14	0.064 (3)	0.066 (2)	0.091 (3)	−0.0256 (19)	0.021 (2)	−0.008 (2)
C15	0.043 (2)	0.086 (3)	0.075 (3)	−0.0148 (18)	0.0161 (19)	0.004 (3)
C16	0.055 (2)	0.071 (3)	0.048 (2)	0.0021 (17)	0.0159 (17)	−0.005 (2)
C17	0.0449 (18)	0.056 (2)	0.045 (2)	−0.0098 (16)	−0.0007 (16)	−0.003 (2)
C18	0.051 (2)	0.065 (2)	0.065 (3)	0.0029 (16)	−0.0041 (18)	0.013 (2)
O3	0.0382 (11)	0.0381 (12)	0.075 (2)	−0.0022 (8)	0.0027 (12)	0.0010 (12)
O4	0.0305 (11)	0.0577 (14)	0.0851 (19)	−0.0060 (9)	0.0001 (12)	0.0028 (14)
C19	0.0303 (15)	0.050 (2)	0.047 (2)	−0.0014 (13)	0.0077 (15)	−0.0055 (18)
C20	0.0472 (19)	0.054 (2)	0.056 (3)	0.0059 (14)	−0.0024 (16)	−0.0032 (19)
C21	0.063 (2)	0.072 (3)	0.069 (3)	0.0117 (19)	−0.001 (2)	0.009 (2)
C22	0.054 (2)	0.114 (4)	0.061 (3)	0.009 (2)	−0.011 (2)	0.015 (3)
C23	0.062 (3)	0.101 (4)	0.067 (4)	−0.019 (2)	−0.012 (2)	−0.011 (3)
C24	0.049 (2)	0.060 (2)	0.070 (3)	−0.0106 (16)	−0.011 (2)	−0.007 (2)
C25	0.0293 (14)	0.0389 (16)	0.054 (3)	−0.0001 (12)	0.0020 (14)	0.0003 (18)
C26	0.0306 (14)	0.0524 (18)	0.059 (3)	0.0022 (13)	−0.0002 (16)	−0.0030 (19)
C27	0.0305 (14)	0.0415 (16)	0.055 (2)	0.0023 (12)	−0.0059 (14)	0.0084 (16)
C28	0.0348 (15)	0.0364 (16)	0.051 (2)	−0.0006 (12)	−0.0049 (14)	0.0046 (16)
C29	0.0334 (15)	0.0346 (16)	0.047 (2)	0.0021 (12)	0.0010 (14)	0.0033 (16)
C30	0.0329 (14)	0.0458 (17)	0.043 (2)	−0.0047 (12)	0.0013 (14)	−0.0108 (16)
C31	0.0549 (19)	0.052 (2)	0.064 (3)	0.0044 (15)	0.0124 (19)	0.0007 (19)
C32	0.050 (2)	0.081 (3)	0.090 (3)	0.0193 (19)	0.016 (2)	−0.009 (3)
C33	0.049 (2)	0.094 (3)	0.073 (3)	−0.009 (2)	0.025 (2)	−0.019 (3)
C34	0.069 (2)	0.076 (3)	0.052 (3)	−0.025 (2)	0.022 (2)	−0.015 (2)
C35	0.0527 (19)	0.0537 (19)	0.042 (2)	−0.0100 (16)	0.0073 (16)	−0.005 (2)
C36	0.0506 (19)	0.052 (2)	0.062 (2)	−0.0052 (14)	−0.0058 (17)	−0.005 (2)

### Geometric parameters (Å, °)

**Table:**

O1—C7	1.424 (3)	C18—H18C	0.9600
O2—C9	1.220 (3)	C18—H18A	0.9600
O1—H1	0.8200	C18—H18B	0.9600
O3—C25	1.421 (3)	C19—C20	1.387 (5)
O4—C27	1.225 (3)	C19—C24	1.380 (5)
O3—H3A	0.8200	C19—C25	1.529 (5)
C1—C6	1.377 (5)	C20—C21	1.370 (6)
C1—C7	1.519 (4)	C21—C22	1.366 (7)
C1—C2	1.382 (5)	C22—C23	1.371 (7)
C2—C3	1.388 (6)	C23—C24	1.382 (7)
C3—C4	1.363 (7)	C25—C29	1.523 (5)
C4—C5	1.364 (7)	C25—C26	1.536 (4)
C5—C6	1.379 (6)	C26—C27	1.500 (5)
C7—C11	1.531 (5)	C27—C28	1.465 (4)
C7—C8	1.551 (4)	C28—C29	1.343 (4)
C8—C9	1.513 (5)	C28—C36	1.505 (5)
C9—C10	1.469 (4)	C29—C30	1.485 (4)
C10—C11	1.337 (4)	C30—C35	1.378 (5)
C10—C18	1.480 (5)	C30—C31	1.389 (5)
C11—C12	1.482 (4)	C31—C32	1.384 (6)
C12—C17	1.382 (5)	C32—C33	1.366 (6)
C12—C13	1.391 (4)	C33—C34	1.370 (6)
C13—C14	1.381 (5)	C34—C35	1.385 (5)
C14—C15	1.359 (6)	C20—H20	0.9300
C15—C16	1.353 (6)	C21—H21	0.9300
C16—C17	1.386 (5)	C22—H22	0.9300
C2—H2	0.9300	C23—H23	0.9300
C3—H3	0.9300	C24—H24	0.9300
C4—H4	0.9300	C26—H26A	0.9700
C5—H5	0.9300	C26—H26B	0.9700
C6—H6	0.9300	C31—H31	0.9300
C8—H8B	0.9700	C32—H32	0.9300
C8—H8A	0.9700	C33—H33	0.9300
C13—H13	0.9300	C34—H34	0.9300
C14—H14	0.9300	C35—H35	0.9300
C15—H15	0.9300	C36—H36A	0.9600
C16—H16	0.9300	C36—H36B	0.9600
C17—H17	0.9300	C36—H36C	0.9600
			
O1···C17	3.212 (4)	C21···H4^viii^	2.8700
O1···O2^i^	2.823 (3)	C22···H4^viii^	3.0300
O2···O1^ii^	2.823 (3)	C24···H3A	2.6900
O2···C17^ii^	3.218 (5)	C27···H3A^iv^	3.0800
O3···C35	3.178 (5)	C27···H18C^iv^	3.0800
O3···O4^iii^	2.861 (3)	C28···H20	2.9700
O4···C35^iv^	3.218 (5)	C29···H20	2.6400
O4···O3^iv^	2.861 (3)	C30···H3A	2.8900
O1···H17	2.8100	C30···H36A	2.7700
O1···H31^v^	2.5900	C31···H36A	2.9800
O1···H6	2.4500	C35···H3A	2.8400
O2···H20^iii^	2.6000	C35···H16^ix^	3.0400
O2···H18B	2.7800	H1···C12	2.8700
O2···H36C^iii^	2.8800	H1···C17	2.8200
O2···H1^ii^	2.0300	H1···H6	2.3200
O3···H13	2.5800	H1···H17	2.5000
O3···H24	2.4700	H1···O2^i^	2.0300
O3···H35	2.7800	H1···C9^i^	3.0500
O4···H36C	2.8800	H1···C6	2.7100
O4···H2^iv^	2.5400	H2···C10	2.9600
O4···H3A^iv^	2.0600	H2···C11	2.6800
O4···H18C^iv^	2.7400	H2···O4^iii^	2.5400
C2···C10	3.528 (5)	H3A···C30	2.8900
C2···C12	3.586 (5)	H3A···C35	2.8400
C2···C9	3.590 (5)	H3A···H24	2.3400
C9···C17^ii^	3.481 (5)	H3A···H35	2.5300
C9···C2	3.590 (5)	H3A···O4^iii^	2.0600
C9···C16^ii^	3.467 (5)	H3A···C27^iii^	3.0800
C10···C2	3.528 (5)	H3A···C24	2.6900
C12···C2	3.586 (5)	H4···C21^x^	2.8700
C13···C18	3.571 (5)	H4···C22^x^	3.0300
C16···C9^i^	3.467 (5)	H6···O1	2.4500
C17···C9^i^	3.481 (5)	H6···H1	2.3200
C17···O2^i^	3.218 (5)	H8A···C2	2.9800
C17···O1	3.212 (4)	H13···O3	2.5800
C18···C13	3.571 (5)	H15···H21^iv^	2.4900
C20···C31	3.525 (5)	H16···C35^xi^	3.0400
C20···C30	3.522 (5)	H17···O1	2.8100
C20···C28	3.543 (5)	H17···H1	2.5000
C27···C35^iv^	3.494 (5)	H18A···C12	2.7900
C27···C34^iv^	3.421 (5)	H18B···O2	2.7800
C28···C20	3.543 (5)	H18C···O4^iii^	2.7400
C30···C20	3.522 (5)	H18C···C27^iii^	3.0800
C31···C36	3.578 (5)	H20···C28	2.9700
C31···C20	3.525 (5)	H20···C29	2.6400
C34···C27^iii^	3.421 (5)	H20···O2^iv^	2.6000
C35···C27^iii^	3.494 (5)	H21···H15^iii^	2.4900
C35···O4^iii^	3.218 (5)	H23···C4^vii^	3.0400
C35···O3	3.178 (5)	H23···C5^vii^	3.0200
C36···C31	3.578 (5)	H24···O3	2.4700
C2···H8A	2.9800	H24···H3A	2.3400
C4···H23^vi^	3.0400	H26A···C20	3.0800
C5···H23^vi^	3.0200	H31···O1^xii^	2.5900
C6···H1	2.7100	H31···C20	3.0700
C9···H1^ii^	3.0500	H34···C17^vi^	2.8900
C10···H2	2.9600	H34···H36B^iii^	2.5400
C11···H2	2.6800	H35···O3	2.7800
C12···H18A	2.7900	H35···H3A	2.5300
C12···H1	2.8700	H36A···C30	2.7700
C17···H1	2.8200	H36A···C31	2.9800
C17···H34^vii^	2.8900	H36B···H34^iv^	2.5400
C20···H31	3.0700	H36C···O4	2.8800
C20···H26A	3.0800	H36C···O2^iv^	2.8800
			
C7—O1—H1	109.00	C10—C18—H18A	110.00
C25—O3—H3A	110.00	C20—C19—C24	117.6 (3)
C2—C1—C6	118.5 (3)	C24—C19—C25	121.5 (3)
C6—C1—C7	121.4 (3)	C20—C19—C25	120.9 (3)
C2—C1—C7	120.1 (3)	C19—C20—C21	121.1 (3)
C1—C2—C3	119.9 (3)	C20—C21—C22	120.8 (4)
C2—C3—C4	120.8 (4)	C21—C22—C23	119.1 (5)
C3—C4—C5	119.6 (5)	C22—C23—C24	120.4 (4)
C4—C5—C6	120.1 (4)	C19—C24—C23	121.0 (4)
C1—C6—C5	121.1 (4)	O3—C25—C19	111.3 (2)
O1—C7—C8	107.6 (2)	O3—C25—C26	107.7 (2)
O1—C7—C1	112.5 (2)	C19—C25—C26	111.7 (3)
C1—C7—C11	111.8 (2)	C19—C25—C29	111.5 (2)
C8—C7—C11	102.8 (2)	C26—C25—C29	103.1 (3)
O1—C7—C11	110.3 (3)	O3—C25—C29	111.2 (3)
C1—C7—C8	111.4 (3)	C25—C26—C27	105.2 (3)
C7—C8—C9	104.4 (3)	O4—C27—C26	126.4 (3)
O2—C9—C10	125.3 (3)	O4—C27—C28	125.0 (3)
O2—C9—C8	125.5 (3)	C26—C27—C28	108.7 (2)
C8—C9—C10	109.3 (2)	C27—C28—C36	122.2 (2)
C11—C10—C18	129.9 (3)	C29—C28—C36	128.6 (3)
C9—C10—C18	121.3 (3)	C27—C28—C29	109.2 (3)
C9—C10—C11	108.8 (3)	C25—C29—C28	112.7 (2)
C7—C11—C10	113.5 (2)	C28—C29—C30	126.1 (3)
C7—C11—C12	121.8 (2)	C25—C29—C30	121.2 (2)
C10—C11—C12	124.7 (3)	C29—C30—C31	120.6 (3)
C11—C12—C13	121.1 (3)	C31—C30—C35	118.5 (3)
C13—C12—C17	118.1 (3)	C29—C30—C35	121.0 (3)
C11—C12—C17	120.8 (2)	C30—C31—C32	120.2 (3)
C12—C13—C14	120.2 (4)	C31—C32—C33	120.4 (4)
C13—C14—C15	120.7 (4)	C32—C33—C34	120.3 (4)
C14—C15—C16	120.0 (4)	C33—C34—C35	119.5 (4)
C15—C16—C17	120.6 (4)	C30—C35—C34	121.2 (3)
C12—C17—C16	120.4 (3)	C19—C20—H20	119.00
C3—C2—H2	120.00	C21—C20—H20	119.00
C1—C2—H2	120.00	C20—C21—H21	120.00
C4—C3—H3	120.00	C22—C21—H21	120.00
C2—C3—H3	120.00	C21—C22—H22	120.00
C5—C4—H4	120.00	C23—C22—H22	121.00
C3—C4—H4	120.00	C22—C23—H23	120.00
C4—C5—H5	120.00	C24—C23—H23	120.00
C6—C5—H5	120.00	C19—C24—H24	120.00
C1—C6—H6	119.00	C23—C24—H24	119.00
C5—C6—H6	119.00	C25—C26—H26A	111.00
C7—C8—H8B	111.00	C25—C26—H26B	111.00
C9—C8—H8A	111.00	C27—C26—H26A	111.00
C9—C8—H8B	111.00	C27—C26—H26B	111.00
H8A—C8—H8B	109.00	H26A—C26—H26B	109.00
C7—C8—H8A	111.00	C30—C31—H31	120.00
C12—C13—H13	120.00	C32—C31—H31	120.00
C14—C13—H13	120.00	C31—C32—H32	120.00
C15—C14—H14	120.00	C33—C32—H32	120.00
C13—C14—H14	120.00	C32—C33—H33	120.00
C16—C15—H15	120.00	C34—C33—H33	120.00
C14—C15—H15	120.00	C33—C34—H34	120.00
C15—C16—H16	120.00	C35—C34—H34	120.00
C17—C16—H16	120.00	C30—C35—H35	119.00
C12—C17—H17	120.00	C34—C35—H35	119.00
C16—C17—H17	120.00	C28—C36—H36A	109.00
C10—C18—H18B	109.00	C28—C36—H36B	109.00
C10—C18—H18C	110.00	C28—C36—H36C	109.00
H18A—C18—H18B	109.00	H36A—C36—H36B	109.00
H18A—C18—H18C	109.00	H36A—C36—H36C	109.00
H18B—C18—H18C	109.00	H36B—C36—H36C	109.00
			
C6—C1—C2—C3	−0.4 (5)	C24—C19—C20—C21	−1.8 (5)
C7—C1—C2—C3	−178.4 (3)	C25—C19—C20—C21	−179.4 (3)
C2—C1—C6—C5	1.0 (5)	C20—C19—C24—C23	1.9 (5)
C7—C1—C6—C5	179.0 (3)	C25—C19—C24—C23	179.5 (3)
C2—C1—C7—O1	−167.8 (3)	C20—C19—C25—O3	−162.5 (3)
C2—C1—C7—C8	71.2 (4)	C20—C19—C25—C26	77.1 (4)
C2—C1—C7—C11	−43.2 (4)	C20—C19—C25—C29	−37.7 (4)
C6—C1—C7—O1	14.3 (4)	C24—C19—C25—O3	20.0 (4)
C6—C1—C7—C8	−106.7 (3)	C24—C19—C25—C26	−100.4 (4)
C6—C1—C7—C11	138.9 (3)	C24—C19—C25—C29	144.8 (3)
C1—C2—C3—C4	−0.6 (6)	C19—C20—C21—C22	0.8 (6)
C2—C3—C4—C5	1.0 (6)	C20—C21—C22—C23	0.1 (6)
C3—C4—C5—C6	−0.3 (6)	C21—C22—C23—C24	0.0 (6)
C4—C5—C6—C1	−0.7 (6)	C22—C23—C24—C19	−1.0 (6)
O1—C7—C8—C9	127.1 (3)	O3—C25—C26—C27	128.2 (3)
C1—C7—C8—C9	−109.1 (3)	C19—C25—C26—C27	−109.3 (3)
C11—C7—C8—C9	10.7 (3)	C29—C25—C26—C27	10.5 (4)
O1—C7—C11—C10	−122.2 (3)	O3—C25—C29—C28	−122.6 (3)
O1—C7—C11—C12	58.8 (3)	O3—C25—C29—C30	57.3 (3)
C1—C7—C11—C10	112.0 (3)	C19—C25—C29—C28	112.6 (3)
C1—C7—C11—C12	−67.1 (3)	C19—C25—C29—C30	−67.6 (3)
C8—C7—C11—C10	−7.6 (4)	C26—C25—C29—C28	−7.4 (4)
C8—C7—C11—C12	173.4 (3)	C26—C25—C29—C30	172.5 (3)
C7—C8—C9—O2	169.2 (3)	C25—C26—C27—O4	170.6 (3)
C7—C8—C9—C10	−11.0 (4)	C25—C26—C27—C28	−10.7 (4)
O2—C9—C10—C11	−173.6 (3)	O4—C27—C28—C29	−174.9 (3)
O2—C9—C10—C18	7.3 (5)	O4—C27—C28—C36	6.8 (5)
C8—C9—C10—C11	6.6 (4)	C26—C27—C28—C29	6.4 (4)
C8—C9—C10—C18	−172.5 (3)	C26—C27—C28—C36	−171.9 (3)
C9—C10—C11—C7	0.9 (4)	C27—C28—C29—C25	0.8 (4)
C9—C10—C11—C12	179.9 (3)	C27—C28—C29—C30	−179.0 (3)
C18—C10—C11—C7	179.9 (3)	C36—C28—C29—C25	179.0 (3)
C18—C10—C11—C12	−1.1 (5)	C36—C28—C29—C30	−0.9 (5)
C7—C11—C12—C13	105.4 (4)	C25—C29—C30—C31	107.3 (3)
C7—C11—C12—C17	−75.6 (4)	C25—C29—C30—C35	−73.4 (4)
C10—C11—C12—C13	−73.6 (5)	C28—C29—C30—C31	−72.9 (5)
C10—C11—C12—C17	105.5 (4)	C28—C29—C30—C35	106.5 (4)
C11—C12—C13—C14	177.9 (4)	C29—C30—C31—C32	179.4 (4)
C17—C12—C13—C14	−1.1 (6)	C35—C30—C31—C32	0.1 (5)
C11—C12—C17—C16	−177.8 (3)	C29—C30—C35—C34	−179.6 (3)
C13—C12—C17—C16	1.3 (5)	C31—C30—C35—C34	−0.2 (5)
C12—C13—C14—C15	0.1 (7)	C30—C31—C32—C33	0.3 (6)
C13—C14—C15—C16	0.7 (7)	C31—C32—C33—C34	−0.6 (7)
C14—C15—C16—C17	−0.5 (6)	C32—C33—C34—C35	0.4 (6)
C15—C16—C17—C12	−0.5 (6)	C33—C34—C35—C30	0.0 (6)

Symmetry codes: (i) *x*−1/2, −*y*+3/2, *z*; (ii) *x*+1/2, −*y*+3/2, *z*; (iii) *x*+1/2, −*y*+1/2, *z*; (iv) *x*−1/2, −*y*+1/2, *z*; (v) *x*, *y*+1, *z*; (vi) −*x*+1, −*y*+1, *z*−1/2; (vii) −*x*+1, −*y*+1, *z*+1/2; (viii) −*x*+1/2, *y*−1/2, *z*+1/2; (ix) −*x*+1/2, *y*−1/2, *z*−1/2; (x) −*x*+1/2, *y*+1/2, *z*−1/2; (xi) −*x*+1/2, *y*+1/2, *z*+1/2; (xii) *x*, *y*−1, *z*.

### Hydrogen-bond geometry (Å, °)

**Table:**

CgD is the centroid of the C19–C25 ring.

**Table:**

*D*—H···*A*	*D*—H	H···*A*	*D*···*A*	*D*—H···*A*
O1—H1···O2^i^	0.82	2.03	2.823 (3)	164.00
O3—H3A···O4^iii^	0.82	2.06	2.861 (3)	164.00
C2—H2···O4^iii^	0.93	2.54	3.430 (5)	162.00
C6—H6···O1	0.93	2.45	2.801 (4)	102.00
C13—H13···O3	0.93	2.58	3.453 (4)	157.00
C20—H20···O2^iv^	0.93	2.60	3.478 (5)	158.00
C24—H24···O3	0.93	2.47	2.803 (6)	101.00
C31—H31···O1^xii^	0.93	2.59	3.478 (5)	161.00
C2—H2···CgC	0.93	2.63	2.918 (4)	99.00
C4—H4···CgD^x^	0.93	2.97	3.750 (5)	142.00
C20—H20···CgF	0.93	2.68	2.957 (4)	98.00

Symmetry codes: (i) *x*−1/2, −*y*+3/2, *z*; (iii) *x*+1/2, −*y*+1/2, *z*; (iv) *x*−1/2, −*y*+1/2, *z*; (xii) *x*, *y*−1, *z*; (x) −*x*+1/2, *y*+1/2, *z*−1/2.
